# Study on the Inhibitory Effects of Three Endophytic *Bacillus* Strains on *Aspergillus flavus* in Maize

**DOI:** 10.3390/metabo15040268

**Published:** 2025-04-11

**Authors:** Siyu Ma, Min Li, Siqi Zhang, Yin Yang, Fengsha Zhu, Xingyu Li, Shahzad Munir, Pengfei He, Pengbo He, Yixin Wu, Yueqiu He, Ping Tang

**Affiliations:** 1 College of Plant Protection, Yunnan Agricultural University, Kunming 650201, China; 2 College of Science, Yunnan Agricultural University, Kunming 650201, China; 3 State Key Laboratory for Conservation and Utilization of Bio-Resources in Yunnan, Yunnan Agricultural University, Kunming 650201, China; 4 Key Laboratory of Agro-Biodiversity and Pest Management of Education Ministry of China, Yunnan Agricultural University, Kunming 650201, China

**Keywords:** *Aspergillus flavus*, *Bacillus*, lipopeptide, inhibition, maize

## Abstract

Background: Maize is easily contaminated by *Aspergillus flavus*, and the aflatoxin produced by *A. flavus* has been classified as a Group 1 carcinogen, for which there are currently no effective control measures. Biological control is regarded as an environmentally friendly and safe approach. Strains ZH179, ZH409, and ZH99 are three bacteria isolated from our laboratory that exhibit antagonistic effects against *A. flavus*. We conducted experiments to investigate their biocontrol efficacy. Results: The experimental results demonstrated that these three strains effectively inhibited *A. flavus* on plates and stored maize seeds. Identification revealed that ZH179 is *Bacillus velezensis*, while ZH409 and ZH99 are *B. amyloliquefaciens*. We also identified lipopeptide synthetase-related genes, including *srfAA*, *srfAD*, *fenA*, *fenB*, *ituA*, *ituB*, *ituD*, *bmyA*, *bmyB*, and *bmyC*, in these three strains. Furthermore, LC-MS analysis confirmed that these strains could produce lipopeptide compounds such as surfactin, fengycin, iturin, and bacillomycin. Using the Oxford cup method, we found that the lipopeptide compounds produced by these strains can inhibit the growth of *A. flavus*. Conclusion: These findings suggest that strains ZH179, ZH409, and ZH99 have good control effects on *A. flavus* during the storage of maize, primarily due to the lipopeptide compounds. This study provides a theoretical basis for using these three strains in the biological control of *A. flavus*.

## 1. Introduction

With the continuous emergence of food safety problems in various countries, the safety of stored grain and oilseeds has gained increasing attention, and fungal contamination has always been a major hidden danger in grain and oil safety. According to statistics, about a quarter of the world’s food crops are contaminated with mycotoxins every year [1], of which *Aspergillus flavus* can infect a variety of food crops, contaminating a variety of food and feed. Moreover, *A. flavus* ranks second only to *A. fumigatus* as a causative agent of Aspergillosis in humans after *A. fumigatus* [2]. Among the more than 300 known mycotoxin-producing fungi, aflatoxins produced by *A. flavus* are the most toxic [3]. At the same time, aflatoxins are associated with strong carcinogenicity, and 4.6% to 28.2% of hepatocellular carcinoma cases globally are due to the ingestion of aflatoxins [4]. Aflatoxins increase not only the economic burden of agricultural production, but also seriously threaten human and animal health. Currently, to reduce aflatoxin contamination, three main types of technical methods are employed: physical, chemical, and biological control measures. However, these physical methods are costly, inefficient, and have high operational risks. Chemical methods carry the risk of drug residues, potentially causing secondary pollution. Owing to its inherent advantages of directness, efficiency, safety, non-toxicity, and cost-effectiveness, the hotspots of research on controlling *A. flavus* contamination in stored grain have shifted towards biological control [5].

Biological control is the core component of green prevention and control technology for plant diseases, which is environmentally friendly, selective, efficient, and safe and does not easily develop resistance [6,7,8]. Among the existing preventive and control measures against *A. flavus* and aflatoxins, the use of natural antagonistic microorganisms for prevention and control is considered a class of methods with great potential, good controllable effects, and prospects [9,10,11,12]. Studies have found that *Bacillus*, *Actinomycetes*, lactic acid bacteria, yeasts, Clausiella, Pseudomonas, and some molds can inhibit the growth and promote toxin degradation of *A. flavus* [13,14,15,16,17,18,19,20]. These biocontrol microorganisms, known as endophytes, commonly colonize various tissues and organs of healthy plants and typically do not cause plant diseases. They are an essential component of the plant micro-ecosystem [21]. Endophytes have been found in seeds, fruits, roots, stems, leaves, and other organs of plants in which seeds, as the reproductive organs of plants, are the main source of genetic transfer of ancestral microbial diversity from generation to generation and can realize the vertical transmission of endophytes [22,23,24,25]. Cottyn et al. isolated 428 strains of seed endophytes from rice seeds harvested in the Philippines, with the main bacteria being *Enterobacteriaceae*, *Bacillus* spp., and *Pseudomonas* spp. About 17 strains of endophytes showed in vitro antifungal activity against *Rhizoctonia solani* or *Pyricularia grisea* [26]. Seed endophytes either directly antagonize fungal spores and mycelia on the seed surface or release antifungal lipopeptides to inhibit fungal growth [27]. Soaking with seed endophytes prior to corn storage can control seedling disease development and post-harvest infection in corn [28].

*Bacillus* species are a common type of plant endophyte known for their role in controlling a variety of fungal diseases. Studies have revealed that the substances responsible for their antifungal activity primarily originate from lipopeptides within their secondary metabolites. These lipopeptides are a class of low molecular weight peptide antibiotics synthesized by non-ribosomal peptide synthetase systems. Their molecular structure exhibits amphiphilic characteristics, consisting of a hydrophilic cyclic oligopeptide chain and a hydrophobic fatty acid chain [29]. In 1948, it was first reported that antimicrobial lipopeptides were isolated from the metabolites of *Bacillus subtilis* [30], and the cyclic lipopeptides were able to inhibit or kill pathogenic microorganisms at lower concentrations effectively and were important substances in the control of plant diseases by *Bacillus* spp. [31]. Iturins and Fengycins secreted by *B. amyloliguefacien* LBM5006 resulted in abnormal development of *R. solani* germ tubes and failure to germinate normally [32]. Bacillomycin D produced by *B. velezensis* HN-2 resulted in crumpling of the surface of the mycelium of *Colletotrichum gloeosporioides*, partial expansion, and the cytoplasm and organelles inside the cell were exuded and formed empty holes [33]. The minimum inhibitory concentration (MIC) of Surfactin crude produced by the *B. safensis* F4 strain against *Staphylococcus aureus* was 0.78 mg/mL [34]. Surfactin causes *Candida* sp. protein and DNA damage, inhibits intracellular reduced glutathione, and causes cell death. In addition, Surfactin damaged the DNA as well as the protein of *Fusarium moniliforme* and reduced GSH content, which prevented maize seeds from being contaminated by *F. moniliforme* [35].

## 2. Materials and Methods

### 2.1. Strains and Culture Condition

The aflatoxin-producing *A. flavus* HO8-B22 strain was isolated from maize seeds in our laboratory. The mycelia attached to sterile filter paper were stored at −80 °C, and the pure culture was inoculated into Potato Dextrose Agar (PDA) medium (containing 200 g glucose, 10 g sugar, and 15 g agar powder per liter of sterile water) for future use. Strains ZH179, ZH99, and ZH409 were isolated from maize, soybeans, and rice seeds, respectively. The three strains were stored at −80 °C in 50% glycerol stock, and their pure cultures were inoculated into Luria-Bertani (LB) medium (containing 10 g tryptone, 5 g yeast extract, and 15 g NaCl per liter of sterile water) for future use.

### 2.2. In Vitro Co-Culture Assay

*A. flavus* HO8-B22 was cultured on a PDA medium in a Petri dish. After the mycelial growth covered the Petri dish, mycelia discs were punched out using a 5 mm diameter puncher. Mycelia discs were placed in the center of the PDA medium plate, and a small amount of bacterial lawn was taken with a sterilized toothpick and inoculated 3 cm away from the *A. flavus* mycelia disc. *A. flavus* was inoculated alone as the mock control. Each treatment was repeated using 3 Petri plates. The inhibiting effect was calculated based on the distance of the transparent zone between the mycelia disc and the bacterial lawn.

### 2.3. Determination of Biocontrol Efficacy on Maize Seeds

The maize seeds’ water content was adjusted to 15% and 20%. After surface sterilization with 1% sodium hypochlorite solution and sterile water, the maize seeds were placed into tissue culture bottles containing 100 g of maize. Then, 1 mL of fermentation broth of antagonistic bacteria (10^7^ spores/mL) was added to each maize bottle. For the control group, 1 mL of sterile culture media was added. After incubation at 28 °C for 7 days, the total mold colony count was determined by referring to the Chinese National Standard of GB/T 13092-2006 [36], and the inhibition rate was calculated as: Inhibition rate (%) = (1 − [Aflatoxin spore concentration in control group]/[Aflatoxin spore concentration in treated group]) × 100.

### 2.4. Identification of Antagonistic Bacterias

The potential antagonistic strains that exhibited significant inhibition in the antagonistic assay were selected for detailed identification based on morphology and molecular methods [37,38]. First, the strains were streaked on the medium with inoculation loops to obtain single colonies, and the morphological characteristics of a single colony were observed under the microscope for identification. Additionally, the growth characteristics of the antagonistic bacterial strain were tested based on physiological and biochemical assays, including Gram staining, aerobic test, indole test, etc. [39,40].

The housekeeping gene *gyrB* was used as the target gene for molecular identification. Briefly, the tested strains were pre-cultured in LB medium for 12 h. Then, the total genomic DNA of isolated strains was extracted using a TIANamp Bacteria DNA isolation kit (TIANGEN^®^ Co., Ltd., Beijing, China) according to the manufacturer’s instructions. Molecular identification of isolated strains was conducted by PCR amplifying the *gyrB* gene. The PCR amplification conditions for the *gyrB* gene were as follows: initial denaturation at 95 °C for 5 min, followed by 30 cycles of denaturation at 95 °C for 30 s, annealing at 56 °C for 30 s, and extension at 72 °C for 90 s, with a final extension at 72 °C for 10 min. For amplification, the primer pair *gyrB*-F (5′-GCCTTGTCGACCACTCTTGA-3′) and *gyrB*-R (5′-AATGGCAGTCAGCCCTTCTC-3′) were used to amplify *gyrB* gene sequences of antagonistic bacteria strains. The PCR amplification products were then sent to the company (TSINGKE^®^ Co., Ltd., Beijing, China) for sequencing, and the obtained sequences were analyzed online using the BLASTN program (http://www.ncbi.nlm.nih.gov/BLAST, accessed on 10 July 2023). A phylogenetic tree was constructed using the Neighbor-Joining method in MEGA11 (Version 11.0.13) software.

### 2.5. Detection of Lipopeptide Biosynthesis Genes of the Three Bacteria

Based on the genes *srfAA*, *srfAD* (coding for Surfactin), *fenA* (coding for Fengycin), *ituB*, *ituD* (coding for Iturin), and *bmyB* (coding for Bacillomycin) identified in the gene sequences of *B. amyloliquefaciens* strains with accession numbers KY051727.1, FJ904932.1, KY051731.1, EU882346.1, MF098754.1, and CP006845.1, primer sequences were designed using SnapGene (Version 7.0.2) software and were then commissioned to be synthesized by Shanghai Jierui Biological Engineering Co., Ltd. (GENEray^®^ Co., Ltd., Shanghai, China). Additional primer sequences of genes *fenB* (coding for Fengycin), *ituA* (coding for Iturin), *bmyA*, and *bmyC* (coding for Bacillomycin) were designed by referring to Qu et al., and all of the primer sequences were shown in Table 1. PCR amplifications were performed with the following conditions: initial denaturation at 94 °C for 4 min, followed by 30 cycles of denaturation at 94 °C for 40 s, annealing at 55 °C for 45 s, and extension at 72 °C for 60 s, with a final extension at 72 °C for 10 min. After the PCR product was subjected to agarose gel electrophoresis, the target fragment was recovered by gelatinization, connected with the pMD18-T carrier, and then the junction product was transformed into *Escherichia coli* TG1. The white spot colonies were selected by the blue-white spot screening method, and the extracted plasmids were then sent to Qingke Xinye Biotechnology Co., Ltd. (TSINGKE^®^ Co., Ltd., Beijing, China) for sequencing.

### 2.6. Characterization of the Antifungal Compounds Using LC-MS

Lipopeptides were the main active substance for the biocontrol of *Bacillus*. After the strain was cultured in the Landy medium [30] for 72 h, the pH of the supernatant obtained by centrifugation was adjusted to 2–3 with 6 mol/L hydrochloric acid. It was then stored in a refrigerator at 4 °C for 12 h and centrifuged again for 2 min at 12,000 r/min. The collected precipitates were dissolved with methanol (about 20 mL in total), and the pH was adjusted to 7 with 6 mol/L NaOH. Finally, the supernatant was dried and concentrated in a water bath to obtain the crude lipopeptide extract.

UPLC-IT-TOF-MS and MS/MS, which were performed according to Li et al. [41], were used to detect the crude lipopeptide extract. Chromatographic separation was carried out using a column (Agilent Zorbax SB-C18 column, 5.0 μm, Ø 4.6 mm × 300 mm, Welch Ultimate, Welch Technology^®^ Co., Ltd., Shanghai, China) with a 1 mL/min flow rate. A mobile phase consisting of methanol (A) and water (containing 0.1% methanoic acid) (B) was used with the following linear gradient: from 0 to 30 min, the ratio of A/B (*v*/*v*) changed from 5:95 to 100:0; from 30 to 40 min, it remained at 100:0. UPLC-IT-TOF-MS performed full scanning in positive mode, and the scanning range was 100 to 1800 *m*/*z*.

### 2.7. Antagonistic Activity Test of Crude Lipopeptide Extract Against A. flavus

The Oxford Cup method was used to detect the antagonistic activity of the crude lipopeptide extract against *A. flavus*. Five milliliters of sterile water were added to the *A. flavus* slant culture grown in a test tube, and the slant was scraped with a sterile inoculation loop to prepare a spore suspension. This suspension was then added to a PDA solid medium cooled to approximately 50 °C, achieving a spore concentration of 10^5^ CFU/mL. After mixing, the medium was poured into Petri dishes with a diameter of 9 cm. Once the medium had cooled and solidified, an Oxford Cup (with an inner diameter of 6 cm) was placed in the center of each Petri dish. Two hundred microliters of the crude lipopeptide extract were pipetted into the Oxford Cup, with methanol and sterile water serving as controls. The cultures were incubated at 28 °C for 72 h, with three replicates set up for each treatment. After incubation, the width of the inhibition zone was measured with the center of the Oxford Cup as the origin.

### 2.8. Statistics

Analysis of the inhibition rate was performed by one-way analysis of variance (ANOVA) using IBM SPSS version 27.0.1 (IBM Corp., Armonk, NY, USA), followed by Duncan’s multiple comparison tests; *p* < 0.05 was considered statistically significant.

## 3. Results

### 3.1. Biocontrol Effect of Three Strains of Bacteria on A. flavus

**Figure 1:**
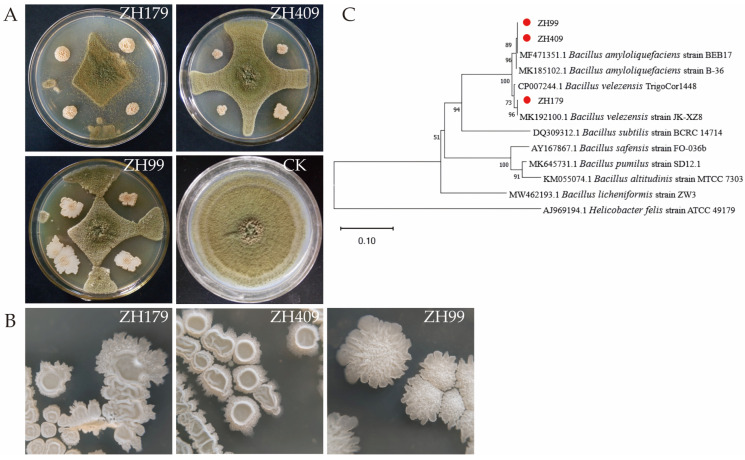
Identification of three strains of biocontrol bacteria. (**A**) The inhibitory effect of 3 bacterial strains on *A. flavus* and the control group. (**B**) Colony morphology of 4 biocontrol strains on LB medium. (**C**) Phylogenetic tree of 3 strains based on *gyrB* gene sequence. A phylogram was generated with MEGA-11 using bootstrap analysis with 1000 replicates and bootstrap support values equal to or greater than 50% are shown at the nodes.

**Figure 2:**
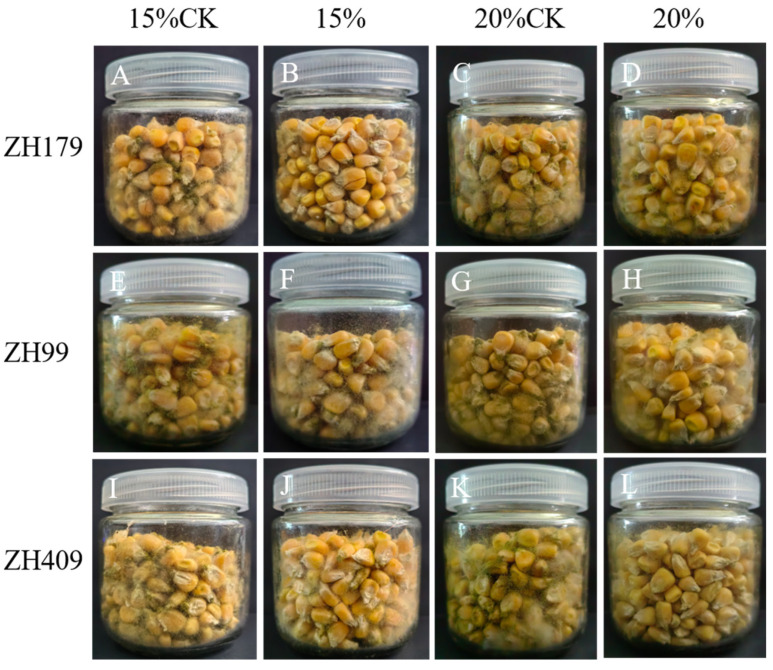
Inhibition of *Aspergillus flavus* by 3 strains of bacteria on maize with different moisture contents. (**A,E,I**): The infection outcomes of *A. flavus* on corn seeds with 15% moisture content in the absence of antagonistic bacteria; (**B,F,J**): The infection outcomes of *A. flavus* on corn seeds with 15% moisture content in the presence of ZH179, ZH99, and ZH409 respectively; (**C,G,K**): The infection outcomes of *A. flavus* on corn seeds with 20% moisture content in the absence of antagonistic bacteria; (**D,H,L**): The infection outcomes of *A. flavus* on corn seeds with 20% moisture content in the presence of ZH179, ZH99, and ZH409 respectively.

Following a seven-day incubation period on PDA media plates, a distinct inhibition zone was observed between the bacterial strains and *A. flavus* (Figure 1). Additionally, maize samples were co-inoculated with the biocontrol bacteria and *A. flavus*. After a 14-day culturing period, significant inhibition of *A. flavus* by the three bacterial strains was observed (Figure 2). Counting the number of *A. flavus* spores on maize seeds, the data revealed that when maize had a water content of 15%, the inhibition ratios of strains ZH179, ZH409, and ZH99 against *A. flavus* were 76%, 67%, and 59%, respectively. In addition, with a 20% water content in maize, the inhibition ratios were 66%, 63%, and 67%, respectively. Notably, under both water content conditions, the inhibition ratios of the three biocontrol bacteria on *A. flavus* in stored maize were remarkably high, exceeding 60% in all cases. Furthermore, compared to the 20% water content, the inhibition ratios were more favorable at the 15% water content. The statistical data on the inhibition rates mentioned above are shown in Table 2.

### 3.2. Identification Results of Biocontrol Strains

Single colony characteristics of three biocontrol strains under a microscope were as follows (Figure 1): The colony edges were not neat, the colony surfaces were rough and opaque, with an uplifted appearance, and the colonies were milky white. The result of the physiological and biochemical reactions of the biocontrol bacteria is shown in Table 3. The *gyrB* gene sequences of the three strains were submitted to the NCBI website. Sequence homology analysis confirmed that strain ZH179 (1088 bp) was *B. velezensis*, while strain ZH409 (1126 bp) and strain ZH99 (1098 bp) were *B. amyloliquefaciens* (Figure 1).

### 3.3. Test Results of the Lipopeptidase Gene

**Figure 3:**
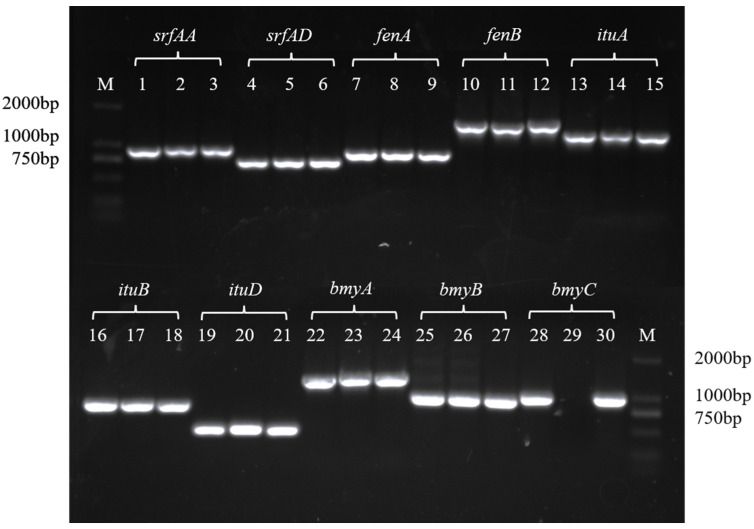
PCR products of genes required for lipopeptide. M: DL 2000 bp marker; 1, 4, 7, 10, 13, 16, 19, 22, 25, 28: *B. velezensis* ZH179; 2, 5, 8, 11, 14, 17, 20, 23, 26, 29: *B. amyloliquefaciens* ZH409; 3, 6, 9, 12, 15, 18, 21, 24, 27, 30: *B. amyloliquefaciens* ZH99.

The lipopeptide synthetase gene in the DNA of strains ZH179, ZH409, and ZH99 was detected, and the PCR products of the expected size were obtained (Figure 3). The specific bands were recycled, cloned for sequencing, and then analyzed using BLASTX in GenBank to further confirm the gene corresponding to lipopeptide synthetase. Sequence analysis revealed high homology between the synthase genes of Fengycin, Surfactin, and Iturin in the three strains and the corresponding synthase genes of strains retrieved from NCBI. These results indicated the presence of genes related to the three major metabolites (Fengycin, Surfactin, and Iturin) in the genomes of strains ZH179, ZH409, and ZH99.

### 3.4. LC-MS Test Results of Crude Lipopeptide Extract

A total of 74 positive ion peaks were detected from the crude lipopeptide extract of strain ZH179, and the test results obtained using LCMS are shown in Table 4. Four surfactant compounds were identified, and their molecular weights were derived from their respective mass–charge ratios. The four compounds were divided into two classes according to molecular weight: one class of 1061.70 and 1075.72, and the other class of 1035.70 and 1049.70. The difference between the molecules of each class was 14 Da, consistent with the molecular weight of methylene (-CH2). This indicated that they might be homologs. Two compounds of Fengycin were identified, the molecular weights of which were 1043.50 and 1057.60, respectively. The molecular weights of Iturin identified were 1043.50 and 1057.60 (with a difference of 14 Da) and homologs. The five compounds of bacitomycin fall into two classes. One class has molecular weights of 1048.50 and 1062.56, while the other has 1044.55, 1058.56, and 1072.58. The difference between the molecules in each class was 14 Da, indicating that it was homologous.

Seventy-seven positive ion peaks were detected from the crude lipopeptide extract of strain ZH409. Four surfactants were identified, including a group of homologs (1061.70, 1075.72). Two Fengycin with molecular weights of 1504.84 and 1532.87 were also identified as homologs. The molecular weight difference between them was 28 Da, which is consistent with the molecular weight of two methylene (-CH2). Both Iturins identified were homologs with molecular weights of 1057.60 and 1071.50, respectively.

From the crude extract of lipopeptides derived from the ZH99 strain, 76 positive ion peaks were detected. Two compounds of Surfactin were identified, with molecular weights of 1061.70 and 1075.72, which were homologs. One compound of Fengycin was identified, with a molecular weight of 1546.89. Five compounds of Iturin were identified and could be classified into two categories: the first category includes compounds with molecular weights of 1043.50, 1057.60, and 1071.50; the second category includes compounds with molecular weights of 1053.51 and 1081.55. The molecular weights within each category differ by integer multiples of 14, indicating that they were homologs. Four compounds of Bacillomycin were identified, and their molecular weights could be divided into two categories: the first category includes compounds with molecular weights of 1034.50 and 1048.50; the second category includes compounds with molecular weights of 1044.55 and 1072.58. The molecular weights within each category differ by 14 Da, indicating that they belonged to two sets of homologs.

When comparing the LC-MS results of the crude lipopeptide extracts from the ZH179, ZH409, and ZH99 strains, we found that all three strains were capable of producing Surfactin, Fengycin, Iturin, and Bacillomycin. Specifically, ZH179 produced four types of Surfactin, two types of Fengycin, two types of Iturin, and five types of Bacillomycin, totaling 13 types of lipopeptides. ZH409 produced four types of Surfactin, two types of Fengycin, two types of Iturin, and three types of Bacillomycin, totaling 11 types of lipopeptides. ZH99 produced two types of Surfactin, one type of Fengycin, five types of Iturin, and four types of Bacillomycin, totaling 12 types of lipopeptides. Among the three strains, ZH179 produced the most diverse range of lipopeptides, followed by ZH99, while ZH409 produced the fewest types.

### 3.5. Biocontrol Effect of Crude Lipopeptide Extract of Biocontrol Bacteria

**Figure 4:**
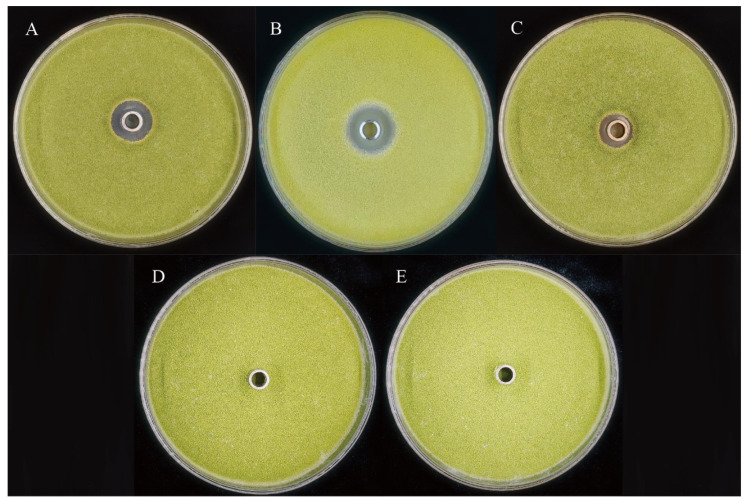
Enhanced inhibitory effect of lipopeptide crude extracts derived from three bacterial strains on *A. flavus*. (**A**) Inhibitory activity of crude lipopeptides derived from strain ZH179 against *A. flavus*. (**B**) Inhibitory activity of crude lipopeptides derived from strain ZH409 against *A. flavus.* (**C**) Inhibitory activity of crude lipopeptides derived from strain ZH99 against *A. flavus*. (**D**) Methanol control group. (**E**) Sterile water control group.

The crude lipopeptide extracts of ZH179, ZH409, and ZH99 were prepared using hydrochloric acid precipitation and methanol extraction methods. The Oxford cup method determined the inhibitory effects of these crude lipopeptide extracts on *A. flavus*. The results are shown in Figure 4: The diameters of the inhibition zones of the crude lipopeptide extracts from strains ZH179, ZH409, and ZH99 against *A. flavus* were 16.50 ± 0.87 mm, 15.83 ± 1.61 mm, and 13.83 ± 0.29 mm, respectively. The crude lipopeptide extracts from all three biocontrol strains exhibited good inhibitory effects on the growth of *A. flavus* hyphae. It was speculated that the lipopeptide compounds produced by these three strains were the primary substances responsible for inhibiting *A. flavus*.

## 4. Discussion

*A. flavus* is a common opportunistic pathogen that is highly susceptible to infecting crops rich in oil, such as maize, peanuts, and cottonseeds [49], and produces highly toxic aflatoxins, which pose a serious threat to human and animal health [50]. *Bacillus* spp. can be isolated from plants, soil, water, and other sources [51]. Due to their short growth cycles and strong stress resistance, they have become a focus of intensive research in fungal disease control in recent years [52,53]. Research has also been conducted on the use of *Bacillus* for the prevention and control of *Aspergillus flavus*. Einloft et al. discovered that *B. safensis* RF69 decreased the growth rate of *A. flavus* by 73.2% and significantly reduced the production of *A. flavus* conidium [54]. Hassan found that the volatile organic compounds produced by *B. licheniformis* BL350-2 could completely inhibit the spore germination of *A. flavus*, reduce the production of toxins, and inhibit the growth of hyphae by up to 88% [55]. Zhao et al. found that Mycosubtilin, produced by *B. subtilis* BS-Z15, was the main substance responsible for inhibiting *A. flavus* [56]. We also obtained similar results; three strains of bio-preventive bacteria with remarkable antagonistic effects against *A. flavus* were screened from numerous endophytes of grain seeds using the plate standoff method. One was *B. velezensis*, and the other two belonged to *B. amyloliquefaciens*. When inoculated into maize seeds with varying moisture contents, these strains exhibited a preventive effect against *A. flavus* that could exceed 60%, thus having the potential to be developed as a mold preventive agent for grain storage.

The *Bacillus* genus is capable of controlling a variety of plant diseases, primarily due to its ability to produce non-ribosomally synthesized lipopeptides [57]. Lipopeptides and lipopeptide-producing microorganisms have been used against bacteria, fungi, oomycetes, and other plant-pathogenic fungi, among which Fengycin, Iturin, and Bacillomycin have good antifungal activity. Surfactin has a broader range of antibacterial and insecticidal activities [58]. Based on the aforementioned related studies, we hypothesized that these three strains of bacteria were capable of producing lipopeptide substances; therefore, we detected their lipopeptide synthetase genes. Our findings revealed that all three strains of bacteria had been detected with the genes: *srfAA*, *srfAD*, *fenA*, *fenB*, *ituA*, *ituB*, *ituD*, *bmyA*, *bmyB*, and *bmyC*, indicating that each of the three strains possessed the potential to produce three types of lipopeptides: Fengycin, Surfactin, and Iturin. To verify whether the three bacterial strains could produce lipopeptide substances with antifungal activity, we extracted their crude lipopeptide extracts and subjected them to detection using liquid chromatography-mass spectrometry (LC-MS) and tandem mass spectrometry (MS-MS) techniques. A single bacterial strain can produce at least one type of lipopeptide substance, while some strains are capable of producing all three types simultaneously [46,59].

In our research, all three tested strains were detected to produce these three types of lipopeptides, indicating their comprehensive antifungal capabilities. Previous studies have also investigated the antifungal mechanisms of these lipopeptides. Krishnan et al. showed that surfactants had a more significant antimicrobial effect on *F. moniliforme* than carbendazim [35]. Tang et al. found that treating *Rhizopus stolonifer* in abundance can cause apoptosis and necrosis of mold cells, which can be used for fruit preservation [60]. Gong et al. found that Iturin A had an excellent inhibitory effect on *F. graminearum*, the source of wheat scab [61]. Zhao et al. found that Bacillomycin D produced by *B. vallismortis* ZZ185 had strong in vitro inhibitory activity against plant pathogens such as *F. graminearum*, *Alternaria alternata*, *Rhizoctonia solani*, *Cryphonectria parasitica,* and *Phytophthora capsici* [62]. In our experiments, we found that the crude extracts of lipopeptides from the three *Bacillus* strains exhibited significant inhibitory effects on *A. flavus*. Our conclusion aligns with previous findings, leading us to deduce that lipopeptides are the primary bioactive components responsible for the inhibitory effects of these three strains. However, the antifungal mechanisms of these strains warrant further investigation in subsequent experiments.

In this study, three *Bacillus* strains antimicrobial lipopeptide synthesis genes were cloned and sequenced. It was revealed that all three strains possessed the potential capacity to synthesize antimicrobial lipopeptides. The crude lipopeptide extracts were obtained through hydrochloric acid precipitation and methanol solubilization, and LC-MS further analyzed the active components in the crude lipopeptide extracts. Although the types of lipopeptide antibiotics produced by the three strains of *Bacillus* sp. were identified, the separation and purification of the individual components in the crude extracts were not conducted. The specific type of lipopeptides that exhibited the main inhibitory activity against *A. flavus* remains to be explored in subsequent studies.

## 5. Conclusions

In conclusion, *Bacillus* sp. ZH179, ZH409, and ZH99 were able to inhibit *A. flavus* spore production in maize seeds. Combining the cloning and sequencing results of lipopeptide synthetase genes and the LC-MS detection results of crude lipopeptide extracts, we found that all three strains could produce multiple lipopeptide compounds. The Oxford cup method also revealed that the crude lipopeptide extracts from these three strains showed clear inhibition zones against *A. flavus*, indicating that lipopeptides were likely the main active substances responsible for these three strains’ inhibition of *A. flavus*. When considering all these results, our research demonstrated that *Bacillus* sp. ZH179, ZH409, and ZH99 exhibited strong inhibitory effects on *A. flavus*, and this study laid the foundation for utilizing these three strains to prevent and control *A. flavus* contamination during maize storage.

## Figures and Tables

**Table 1:** Primer sequences for lipopeptide-related gene detection.

Antimicrobial Lipopeptides	Target Gene	Primers Name	Primers Sequences (5′→3′)	Size/bp
Surfactin	*srf AA*	*srfAA*-F	GCTTGTTAGGTCATGTGCGCAAG	722
		*srfAA*-R	CTTTGCTGAGTCAGGAGCACATTCG	
	*srf AD*	*srfAD*-F	CCTAAGGAAGGAACATCCGAAGC	606
		*srfAD*-R	GGACGGGTGATTGAATCATGTGAG	
Fengycin	*fenA*	*fenA-F*	GGTTGACTCCCATTATCCTGAGGAAC	740
		*fenA-R*	GAACACCGATCGGCACATCATCTT	
	*fenB*	*fenB*-F	CTATAGTTTGTTGACGGCTC	1600
		*fenB*-R	CAGCACTGGTTCTTGTCGCA	
Iturin	*ituA*	*ituA*-F	ATGTATACCAGTCAATTCC	1047
		*ituA*-R	GATCCGAAGCTGACAATAG	
	*ituB*	*ituB-F*	CGATCGGCTGGATTTGATGGTG	722
		*ituB-R*	GCTTCATGATGCGGATGCAGAC	
	*ituD*	*ituD-F*	GCAGGCCATAGCTTAGGCGAATATTC	361
		*ituD-R*	AGGCGGATCGTATCATCGAACTG	
Bacillomycin D	*bmyA*	*bmyA*-F	AAAGCGGCTCAAGAAGCGAAACCC	1200
		*bmyA*-R	CGATTCAGCTCATCGACCAGGTAGGC	
	*bmyB*	*bmyB-F*	CATGCAAATCCTGCATCAAGTCGTG	816
		*bmyB-R*	CGCACAATTGATTCAAGCAGAGCTG	
	*bmyC*	*bmyC*-F	GAAGGACACGGCAGAGAGTC	875
		*bmyC*-R	CACTGATGACTGTTCATGCT	

**Table 2:** Inhibition of 3 biocontrol strains on *A. flavus* on maize seeds.

Strain No.	15% Water Content		20% Water Content	
	Mean Spore Number (×10^5^ CTU/mL)	Inhibition (%)	Mean Spore Number (×10^5^ CTU/mL)	Inhibition (%)
ZH179	3.20 ± 0.20 ^a^	76.3	9.53 ± 1.03 ^a^	66.0
ZH99	4.47 ± 0.32 ^c^	66.9	10.3 ± 1.06 ^a^	63.2
ZH409	5.53 ± 0.57 ^b^	59.0	9.33 ± 0.81 ^a^	66.7

Lowercase letters indicate significant inhibitory effects (*p* < 0.05).

**Table 3:** Physiological and biochemical reactions of 3 bacterial strains.

Test Items	ZH99	ZH179	ZH409
Methyl red	-	-	-
Starch hydrolysis	+	+	+
V-P	-	-	-
Fermentation of sugars or indoleacetic acid	+	+	+
indole	+	+	+
Gelatin liquefaction	+	+	+
Gram stain	+	+	+
hydrogen sulfide	-	-	-
Utilization of citrate	+	+	+
4% KOH reaction	-	-	-
3% H_2_O_2_ reaction	+	+	+
Utilization of malonate	-	-	-
Urease reaction	+	+	+

Note: “+” means positive reaction; “-” means negative reaction.

**Table 4:** Qualitative analysis results of lipopeptide crude extracts of three strains of Bacillus by LC-MS.

Strains	Lipopeptide Antibiotic Class	Mass-to-Charge Ratio (*m*/*z*)	Calculate the Molecular Weight (Da)	Ionic Type	Relative Content (%)	Literature
ZH179	Surfactin	1076.67	1075.72	[M+H]^+^	2.34	[42]
	Surfactin	1062.62	1061.70	[M+H]^+^	0.83	[42]
	Surfactin	1050.60	1049.70	[M+H]^+^	0.22	[42]
	Surfactin C (C15)	1058.66	1035.70	[M+Na]^+^	2.59	[42]
	Fengycin	767.41	1532.87	[M+2H]^2+^	0.92	[43]
	Fengycin	766.40	1530.90	[M+2H]^2+^	5.40	[43]
	Iturin A2	1043.50	1044.66	[M+H]^+^	0.99	[44]
	IturinB (C15)	1058.61	1057.60	[M+H]^+^	0.43	[45]
	Bacillomycin L (C15)	525.28	1048.50	[M+2H]^2+^	2.42	[46]
	Bacillomycin L	1063.56	1062.56	[M+H]^+^	0.93	[46]
	Bacillomycin D	1067.53	1044.55	[M+Na]^+^	13.83	[47]
	Bacillomycin D	1081.55	1058.56	[M+Na]^+^	7.53	[47]
	Bacillomycin D	1095.57	1072.58	[M+Na]^+^	4.69	[47]
ZH409	Surfactin	1062.66	1061.70	[M+H]^+^	0.89	[42]
	Surfactin	1076.68	1075.72	[M+H]^+^	2.40	[42]
	Surfactin	1065.53	1064.70	[M+H]^+^	3.04	[42]
	Surfactin	1044.65	1021.67	[M+Na]^+^	3.22	[42]
	Fengycin	753.45	1504.84	[M+2H]^2+^	1.41	[43]
	Fengycin	767.41	1532.87	[M+2H]^2+^	11.08	[43]
	IturinB (C15)	1058.66	1057.60	[M+H]^+^	2.07	[45]
	Iturin A6	1072.69	1071.50	[M+H]^+^	0.55	[48]
	Bacillomycin L (C15)	525.28	1048.50	[M+2H]^2+^	1.94	[47]
	Bacillomycin L	532.36	1062.56	[M+2H]^2+^	0.23	[46]
	Bacillomycin D	537.32	1072.58	[M+2H]^2+^	0.05	[47]
ZH99	Surfactin	1062.66	1061.70	[M+H]+	1.99	[42]
	Surfactin	1076.68	1075.72	[M+H]^+^	4.30	[42]
	Fengycin	774.65	1546.89	[M+2H]^2+^	0.07	[43]
	Iturin A2	1044.66	1043.50	[M+H]^+^	0.89	[45]
	Iturin B (C15)	1058.62	1057.60	[M+H]^+^	0.23	[45]
	Iturin A6	536.28	1071.50	[M+2H]^2+^	0.22	[48]
	Iturin C	1054.52	1053.51	[M+H]^+^	7.40	[45]
	Iturin C	1104.67	1081.55	[M+Na]^+^	1.20	[45]
	Bacillomycin L (C14)	518.40	1034.50	[M+2H]^2+^	0.26	[46]
	Bacillomycin L (C15)	1049.60	1048.50	[M+H]^+^	0.66	[47]
	Bacillomycin D	1067.53	1044.55	[M+Na]^+^	9.65	[47]
	Bacillomycin D	1095.57	1072.58	[M+Na]^+^	3.53	[47]

## Data Availability

The original contributions presented in this study are included in the article. Further inquiries can be directed to the corresponding author.
